# Fracture Resistance and Microleakage around Direct Restorations in High C-Factor Cavities

**DOI:** 10.3390/polym14173463

**Published:** 2022-08-25

**Authors:** Emese Battancs, Tekla Sáry, Janka Molnár, Gábor Braunitzer, Máté Skolnikovics, Árpád Schindler, Balázs Szabó P., Sufyan Garoushi, Márk Fráter

**Affiliations:** 1 Department of Operative and Esthetic Dentistry, Faculty of Dentistry, University of Szeged, H-6720 Szeged, Hungary; 2 DicomLAB Dental Ltd., H-6726 Szeged, Hungary; 3 Schindler Dent Ltd., H-6400 Kiskunhalas, Hungary; 4 Department of Food Engineering, Faculty of Engineering, University of Szeged, H-6725 Szeged, Hungary; 5 Department of Biomaterials Science and Turku Clinical Biomaterials Center—TCBC, Institute of Dentistry, University of Turku, FI-20520 Turku, Finland

**Keywords:** direct restoration, short fibre-reinforced composite, occlusal filling, fracture resistance, microleakage, high C-factor, bulk-fill

## Abstract

The aim of this research was to evaluate the mechanical impact of different direct restorations in terms of fracture resistance, and subsequent fracture pattern, in occlusal high C-factor cavities. Furthermore, the adaptation of different direct restorations in the form of gap formation was also evaluated. Seventy-two intact mandibular molars were collected and randomly distributed into three groups (*n* = 24). Class I occlusal cavities with standardized dimensions were prepared in all specimens. After adhesive treatment, the cavities were restored with direct restorations utilizing three different materials. Group 1: layered conventional packable resin composite (Filtek Ultimate), Group 2: bulk-fill resin composite (SDR), Group 3: bulk-fill short fibre-reinforced composite (SFRC; everX Posterior) covered with packable composite occlusally. Half of the restored specimens underwent static load-to fracture testing (*n* = 12/group), while the rest underwent sectioning and staining for microleakage evaluation and gap formation analysis. Fracture patterns were evaluated visually among the mechanically tested specimens. The layered composite restoration (Group 1) showed significantly lower fracture resistance compared to the bulk fill groups (Group 2, *p* = 0.005, Group 3, *p* = 0.008), while there was no difference in fracture resistance between the other groups. In terms of gap formation values, the layered composite restoration (Group 1) produced significantly higher gap formation compared to the bulk-fill groups (Group 2, *p* = 0.000, Group 3, *p* = 0.000). Regarding the fracture pattern, SFRC (Group 3) produced the highest number, while SDR (Group 2) produced the lowest number of repairable fractures. The use of bulk-fill resin composite (fibre or non-fibre-reinforced) for occlusal direct restorations in high C-factor cavities showed promising achievements regarding both fracture resistance and microleakage. Furthermore, the use of short fibre-reinforced bulk-fill composite can also improve the fracture pattern of the restoration-tooth unit. Bulk-fill materials provide a simple and effective solution for restoring and reinforcing high C-factor occlusal cavities.

## 1. Introduction

Today, resin composite materials are the primary choice for direct restorations in the posterior dentition, and clinical studies reported high clinical performance and durability [1,2,3]. Since the introduction of resin composites more than 50 years ago, they have undergone constant development [4]. Some material-related issues, however, still remain, and these lead to problems for both the operator and the patient. One of these issues is the polymerization shrinkage of the restorative material. Polymerization shrinkage generates stress within resin composites, at the interface between the composite restoration and the tooth substance, as well as within the tooth structure [4]. This can lead to internal and marginal gaps, micro-cracking of either the restorative material and tooth structure (or both), and marginal stain cuspal movement [5], which can, in turn, result in restoration loss [6]. Conversely, in anterior restorations, shrinkage issues lead to fewer consequences, although remaining aesthetic concerns [7,8]. One possible solution to minimize these negative effects of polymerization shrinkage and related stress is utilizing an incremental technique when placing the composite material. In this technique, resin composites are applied in horizontal or oblique increments of a maximum thickness of 2 mm. As each increment is light-cured separately, the technique allows adequate light penetration to cure the material [9]. This procedure is thought to reduce the final volumetric shrinkage of the material, and thus minimize internal gap formation [10]. Furthermore, it also decreases the C-factor (the ratio of bonded surface to unbonded free surface) [9,11]. However, the incremental technique is complex, it increases chair time and voids may be included between the increment layers [10,11].

Due to the clear demand for simplified application and reduced application time, so-called ‘‘bulk-fill’’ resin composites have been developed for restoring Class I and Class II posterior cavities [4]. Bulk-fill resin composites can be applied in a single increment of 4–5 mm thickness, depending on the product [12]. The mechanical properties of bulk-fill resin composites promote lower polymerization shrinkage stress, better stress distribution, good adaptation to the cavity walls and high fracture resistance [13,14]. However, this clearly depends on the specific material [5,13]. To ease application and adaptation, flowable bulk-fill composites with lower filler content have been brought to the market. The first marketed flowable bulk-fill composite was SDR (Dentsply Sirona, Wien, Austria), which contains a stress-relieving additive in the monomer matrix [1,15]. So far, several studies have confirmed that the polymerization shrinkage stress of SDR is lower than that of other low-viscosity (conventional and bulk-fill) composites or conventional high-viscosity composites [16,17,18].

The other challenge with resin composite materials is their inadequate fracture toughness compared to dentine’s [19]. Fracture toughness describes the damage tolerance of the material and can be considered as a measure of fatigue resistance, which predicts structural performance [20,21]. Most likely due to this deficiency, a resin composite direct filling is not the best solution to reinforce deep cavities in molar teeth [22,23,24]. A solution to this problem has been proposed to be the use of short fibre-reinforced composites (SFRC) to substitute the missing dentine in direct or indirect restorations [23,25,26]. Incorporation of fibres into dental resin composites have been studied extensively and has shown superior mechanical performance compared to non-fibre containing resin composite restorative materials [27,28,29,30,31,32]. Bijelic-Donova et al. showed that SFRC had a significantly higher fracture toughness (2.4 MPa m^1/2^) and fatigue limit than conventional PFC resins (range: 0.9–1.1 MPa m^1/2^) [33]. With its high fracture toughness and other unique features, SFRC can work as a stress-absorbing layer within the restoration [24,34]. Furthermore, as SFRC is quite transparent and short fibres can scatter the light, SFRC can and should be used as a bulk-fill material with a curing depth up to 5 mms [35,36,37]. However, due to its high viscosity, internal adaptation and void formation remain as potential challenges.

The aim of our study was to evaluate the internal adaptation and fracture resistance of direct restorative fibre-reinforced and non-fibre-reinforced composite materials in high C-factor cavities.

## 2. Materials and Methods

The study procedures were approved by the Regional Human Biomedical Research Ethics Committee of the University of Szeged. The study procedures conformed to the Declaration of Helsinki in all respects.

Seventy-two mandibular molars extracted for periodontal reasons were selected for the investigation. The teeth were randomly divided into three test groups (Groups 1–3, *n* = 24). The teeth had similar morphological parameters in terms of their coronal dimensions (mesio-distal width, bucco-lingual width, coronal height, allowing a maximum of 10% deviation from the measured mean). The following exclusion criteria were applied: caries, former endodontic procedures, posts or other coronal restorations, any visible crack or fracture. The freshly extracted molar teeth were kept in 5.25% NaOCl for 5 min before being preserved at room temperature in 0.9% saline solution until use. All teeth were used within 8 weeks of extraction. Hand scalers were used to remove the soft tissue covering the root surface.

### 2.1. Cavity Preparation

Class I occlusal cavities of standardized size were prepared in the central area of the occlusal surface of each tooth, with the following parameters: the length of the cavity was 5 mm (mesio-distal dimension), its width was 4 mm (bucco-lingual dimension) and its depth was also 4 mm.

The preparation was performed under water cooling, using a round end parallel diamond bur (883H.806.314146.544 FG, Hager & Meisinger, Neuss, Germany). The cavity walls were prepared parallel to the axis of the tooth. The cavity dimensions were constantly evaluated with a 15 UNC periodontal probe (American Eagle Probe UNC 15, American Eagle Instruments, Missoula, MT, USA). The depth of the cavity was measured from cusp tip to cavity floor in a way that the entire length of the probe was touching the cavity wall while the measurement was taken. Restoration took place in the same session, after cavity preparation. All used restorative materials with their main physical properties are listed in Table 1 and Table 2.

### 2.2. Restoration

**Figure 1:**
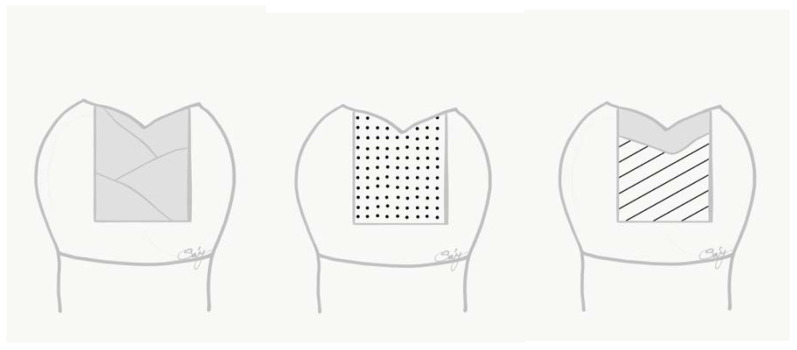
Schematic figure representing the test groups (from left to right). Group 1: layered conventional packable composite filling; Group 2: bulk-fill composite filling; Group 3: bulk-fill SFRC and conventional packable composite occlusal coverage.

All specimens underwent the same adhesive treatment. Enamel was selectively acid-etched with 35% phosphoric acid for 15 s, washed with water and air dried. A one-step, self-etch, universal adhesive system (G-Premio Bond, GC Europe, Leuven, Belgium) was used for bonding according to the manufacturer’s instructions. Extra adhesive was eliminated by suction drying (Evacuation Tip, Starryshine, Anaheim, CA, USA) within 0.5 cm from the cavity. The adhesive was light-cured for 20 s with an Elipar Deep Cure-L LED light (3M, St. Paul, MN, USA). The average power density of the light source, measured with a digital radiometer (Jetlite light tester; J. Morita USA Inc. Irvine, CA, USA) prior to the bonding procedure, was 1200 ± 150 mW/cm^2^. Teeth were restored with three different direct restorative materials and related techniques as follows (Figure 1): 

Specimens in Group 1 were restored with a conventional packable composite material (Filtek Ultimate-A2 enamel shade and A3 dentine shade, 3M) layered obliquely (in maximum 2 mm increments). Each increment was light-cured for 20 s.

Group 2 was restored with a flowable bulk-fill composite material (SDR, Dentsply Sirona) as bulk-fill. The material was used according to the manufacturer’s instructions. The material was dispensed directly into the cavity using slow, steady pressure, in a single increment, and light-cured for 20 s.

The cavities of Group 3 were restored with packable SFRC (everX Posterior, GC Europe) applied as bulk-fill. The material was placed in single increment, observing the anatomy of dentine, leaving 1.5 mm occlusally for the final composite layers. The SFRC was light-cured from the occlusal surface for 40 s. The final layer was conventional packable composite material (Filtek Ultimate-A2 enamel shade). This layer was light-cured for 20 s.

All restorations were finished with a fine granular diamond bur (FG 7406-018, Jet Diamonds, Kerr, USA and FG 249-F012, Horico, Germany) and aluminium oxide polishers (One GlossPS Midi, Shofu Dental Gmbh, Ratingen, Germany). After the restorative procedures, mechanical testing was carried out on 12 teeth from each group (*n* = 36) and a further 12 teeth from each restored group (*n* = 36) underwent sectioning and microleakage and gap formation analysis.

### 2.3. Mechanical Testing 

Mechanical testing was performed according to the protocol used in our previous studies performed on molar teeth [20,22,23,38]. To simulate the periodontal ligament, the root surface of each tooth was coated with a layer of liquid latex separating material (Rubber-Sep, Kerr, Orange, CA) prior to embedding. The specimens were embedded in methacrylate resin (Technovit 4004, Heraeus-Kulzer, Hanau, Germany) at 2 mm from the cementoenamel junction (CEJ) to simulate the bone level. The restored specimens were then submitted to static load-to-fracture testing (Lloyd R1000, Lloyd Instruments Ltd., Fareham, UK) at a crosshead speed of 2 mm/min. Load was applied using a ball-shaped stainless steel stylus of 6 mm in diameter, positioned at the centre of the occlusal surface of the tooth between the buccal and oral cusps. A force vs. extension curve was dynamically plotted for each specimen. Fracture threshold was defined as the load at which the tooth-restoration complex exhibited the first fracture, resulting in a peak formation on the extension curve, and it was recorded in Newtons (N).

### 2.4. Microleakage Testing and Gap Formation Evaluation

Preparation of the specimens for microleakage testing was performed according to the protocol of Yamazaki et al. [30]. The specimens were dipped in artificial saliva at 37 °C for 24 h, and the roots were covered with two coats of nail polish till reaching 1 mm around the margins of the restoration After the varnish had dried, the apical end of the tooth was dipped in heated wax. Subsequently, specimens were placed in 1% methylene blue for 24 h. An acrylic resin (Duracryl ™ Plus, Spofa Dental, Kerr, Jičín, Czech Republic) was used for embedding the later stained specimens. The specimens were stored at 35 °C for 30 min at 3 bar pressure using a polymerizer. The teeth were sectioned longitudinally, in a sagittal plane into 2 slabs, along the central fissure with a wet trimmer (MT3-Renfert, Hilzingen, Germany). The sectioned specimens were evaluated using a DCM-310 digital camera (Scopetek, Hangzou, China) attached to a stereo microscope at 40× magnification (Carl Zeiss, JENA, Germany) with Plan Achromat 4×/0.10 objective and ScopePhoto (Scopetek). Each sectioned specimen was divided into 4 sites, and dye penetration along the cavity walls was analysed on the sectioned sample. The degree of microleakage according to the depth of dye penetration was assessed using a 4-grade scale as a modification of previous scoring systems [39] (Table 3).

After dye penetration evaluation, internal gap formation and the actual size of the gap was measured along the cavity wall—restoration interface. In each of the four sites, measurement was taken on 20 points along the interface (80/sectioned specimens).

## 3. Results

**Figure 2:**
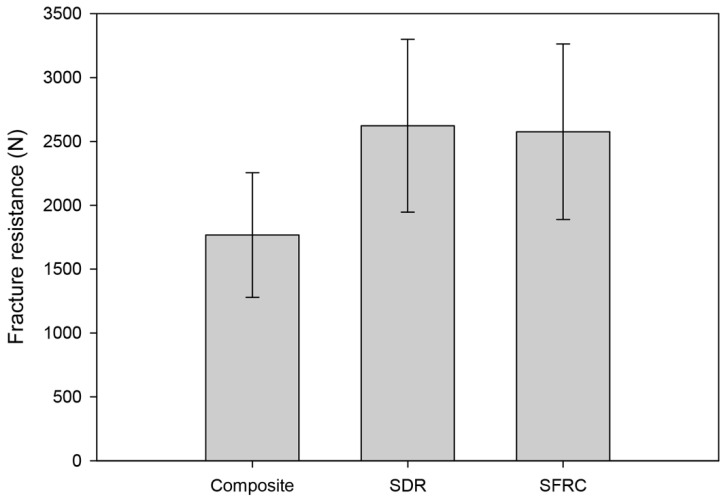
Column plots of fracture resistance by group (group mean +/− SD).

Figure 2 displays the fracture thresholds for the different study groups. The layered composite filling (Group 1) showed significantly lower fracture resistance compared to the bulk-fill groups (Group 2, *p* = 0.005, Group 3, *p* = 0.008), while there was no difference in fracture resistance between the other groups (Figure 2). Thus, the first null hypothesis was rejected.

Regarding the fracture pattern, cavities restored with SFRC (Group 3) managed to show the highest number (dominantly restorable), while teeth restored with SDR (Group 2) presented the lowest number (dominantly non-restorable) of repairable fractures (Table 4). Therefore, the null hypothesis regarding fracture patterns was also rejected.

**Figure 3:**
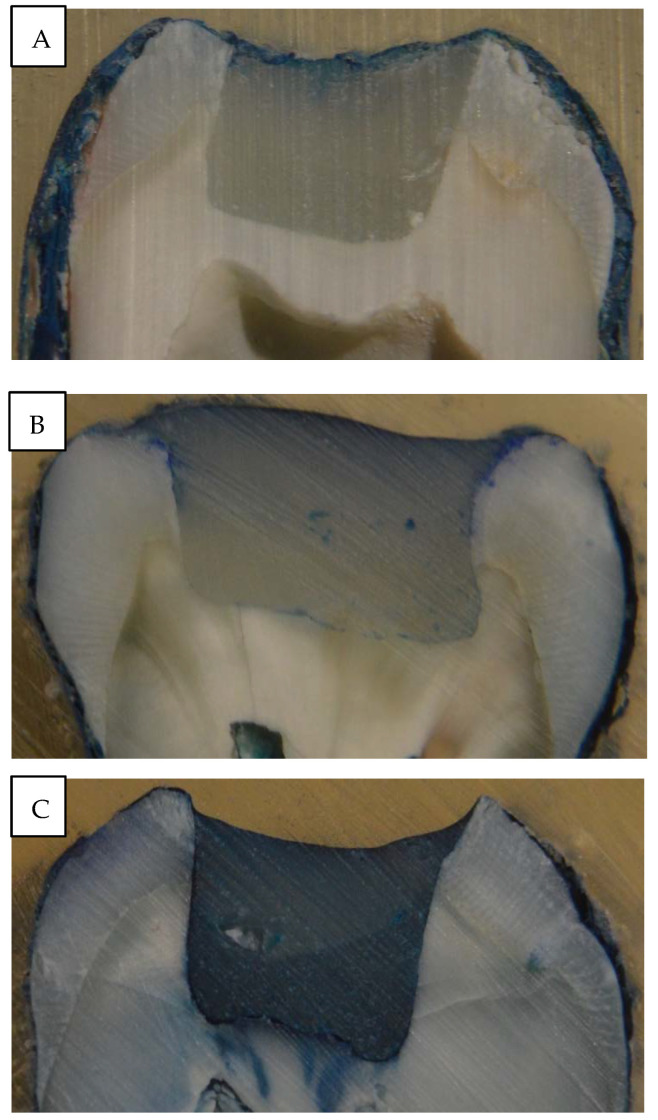
Pictures (**A**–**C**) of the sectioned specimen after dye penetration analysis. Picture (**A**) showed minimal to no marginal staining, while the other pictures show moderate (Picture (**B**)) to deep (Picture (**C**)) penetration of the dye, starting from the margins.

Regarding microleakage testing, Group 2 showed dominantly shallower penetration along the interface, while the rest of the groups (Group 1 and 3) produced dominantly deeper dye penetration reaching deeper parts of the cavity and the interface (Table 5 and Figure 3).

**Figure 4:**
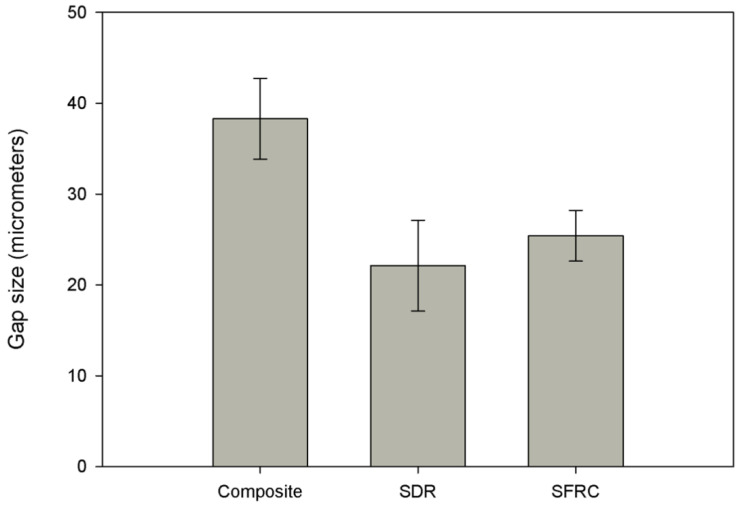
Column plots of gap size by group (group mean +/− SD).

In terms of gap formation values at the bonding interface, layered composite filling (Group 1) produced significantly higher gap formation compared to the bulk-fill groups (Group 2, *p* = 0.000, Group 3, *p* = 0.000) (Figure 4). Therefore, the third null hypothesis was also rejected. Again, the two bulk-fill groups (Group 2 and 3) did not differ in gap formation along from each other along the restoration-cavity interface.

## 4. Discussion

The two main failure types of direct posterior composite restorations in clinical settings are secondary caries and bulk fractures [3,40]. The former is related to early gap formation and subsequent degradation of the interface between the direct restoration and the cavity walls [10]. As already shown by many, polymerization shrinkage and subsequent stress development can be a reason behind marginal gap formation, leading to marginal discoloration, nanoleakage and therefore secondary caries [4,41,42]. The magnitude of the polymerization-related stress depends upon the cavity configuration (C-factor), and the physical characteristics of the composite material, namely its elastic modulus and polymerization conversion rate [43]. When cavities with high C-factor are filled with large amounts of composite material, the integrity of the bond interface is at risk and thus also the longevity of the restoration [44]. This was emphasized by Han et al. who pointed out that in high C-factor cavities the internal adaptation was inferior and the prevalence of imperfect margins was higher compared to low C-factor cavities [45]. The deleterious effects of high C-factor cavities are also visible when measuring microtensile bond strength, compared to low C-factor cavities [46]. Among vital posterior cavities, C-factor is the highest in Class I occlusal cavities. As restoring high C-factor cavities with direct composite restorations could be problematic from a mechanical and gap formation point of view [47], high C-factor Class I occlusal cavities were tested in our study. As the results from the static load-to-fracture tests show, cavities restored with bulk-fill materials (Groups 2 and 3) showed significantly higher load bearing capacity compared to the layered composite filling group (Group 1), at *p* < 0.001 for both groups. This is in accordance with the findings of Rosatto et al., where SDR was characterized by higher fracture resistance than layered composite fillings [14]. However, our present findings are in contrast with the findings of other studies regarding fracture resistance [48,49,50,51]. Bonilla et al. and Rosa de Lacerda et al. did not manage to show any difference in fracture resistance between teeth restored with a bulk-fill composite and a layered composite filling. Of note, neither of these studies used SDR as a bulk-fill composite [48,49].

Bulk-fill composites may vary according to their composition, the specific monomer and photoinitiator system they use, their consistency, and whether they need to be capped with conventional composite or not. It is thus possible that the difference between the results of the cited studies and our results presented in this study stems from the fact that the other studies used materials of different compositions. De Assis et al. did not find difference in fracture strength when comparing cavities restored with SDR or a layered conventional composite filling [50]. Al-Nahedh et al. even found SDR to be inferior to layered composite fillings in terms of fracture resistance in Class II cavities [51]. The same authors also pointed out that when SDR was capped with conventional composite occlusally, the fracture resistance of the resulting restoration was not different from that of a layered composite restoration. It is important to highlight that due to wear and aesthetic properties, clinically SDR is recommended to be covered with conventional composite except in conservative occlusal cavities.

In our study, SFRC restorations showed significantly higher fracture resistance compared to layered composite fillings in Class I cavities (*p* = 0.008). This is in line with the findings of Molnár et al. [24]. SFRC is a dental restorative composite intended to be used in high stress-bearing areas as a dentine replacement material [52,53,54]. Mechanical testing has shown that the load-bearing capacity, flexural strength and fracture toughness of SFRC is superior in comparison with conventional composite materials. As SFRC can be light cured up to 5 mm [55], it was used in a bulk-fill manner in our study.

In terms of fracture pattern, SFRC (Group 3) showed the highest number and also showed dominantly repairable fractures. This shows that by the use of SFRC, a more favorable fracture profile can be reached as compared to conventional composite restorative materials, and this is in line with our previous findings [20,23,38]. The explanation most probably lies in the obvious difference in fracture toughness between reinforced and non-reinforced composites [20,25]. Previous studies have shown that SFRC can re-direct and stop crack propagation within materials [21,25,56]. When non-fibre-reinforced composites were used for restoring cavities in the posterior region, both favourable and unfavourable fractures occurred. SDR restorations were predominantly characterised by non-repairable fractures. This is in line with previous studies on the usual fracture pattern when utilising non-fibre-reinforced composites [20,22,23,38]. As shown by Molnár et al. via fractography, the brittleness of the conventional composite materials generates the bulk fracture, which propagates easily through the whole thickness of the restoration [24].

Regarding the adaptation and subsequent gap formation of the restorative materials, bulk-fill materials (Groups 2 and 3) in our study were characterised by significantly smaller gaps at the bonded interphase compared to the layered composite filling (Group 1), at *p* < 0.001 for both groups. Bulk-fill materials have been developed to eliminate the need for incremental layering [10]. So far, bulk-fill restorations seem to perform like conventional restorations in terms of marginal adaptation, physico-mechanical properties, fatigue resistance and the tendency to induce cuspal deflection [49,57,58,59]. The favorable results for SDR may be explained by SDR generating less polymerization shrinkage stress, possibly as a result of containing a ‘‘polymerization modulator’’ [50,60], which supposedly counteracts polymerization stress through lower polymerization rate [50,61]. Our findings regarding SDR in terms of gap formation are in line with the findings of Peutzfeldt et al. [4] and Al-Nahedh et al. [51]. The explanation for this observation could well be the flowable consistency of SDR [62]. The appearance of flowable bulk-fill materials will probably make the bulk-filling technique even more popular with clinicians as several advantages have been recorded, including low polymerization shrinkage and stress, reduced cuspal deflection and improved self-leveling ability [16,63]. Although flowable composites generally shrink more than conventional paste-like composites, due to their higher amount of resin matrix [64], the subsequent shrinkage stress remains low in comparison [46,65]. Our findings are in contrast with those of Thongbai-On et al. [66] or Park et al. [16] who did not find significant difference between SDR and layered composite filling in terms of gap formation along the interphase. It must be noted that both of the cited studies investigated Class II MO/MOD cavities, which can be an explanation for the different outcomes. In our study, bulk-fill SFRC was characterised by significantly fewer gaps than layered composite restorations (*p* = 0.000). This might be attributed to the unique fibre content and anisotrophicity of the SFRC material. Garoushi et al. pointed out that the orientation of the reinforcing fibres in anisotropic materials has a major influence on polymerization shrinkage: as it is controlled in the direction of the fibres, it is never homogeneously distributed in all directions [35,55].

**Figure 5:**
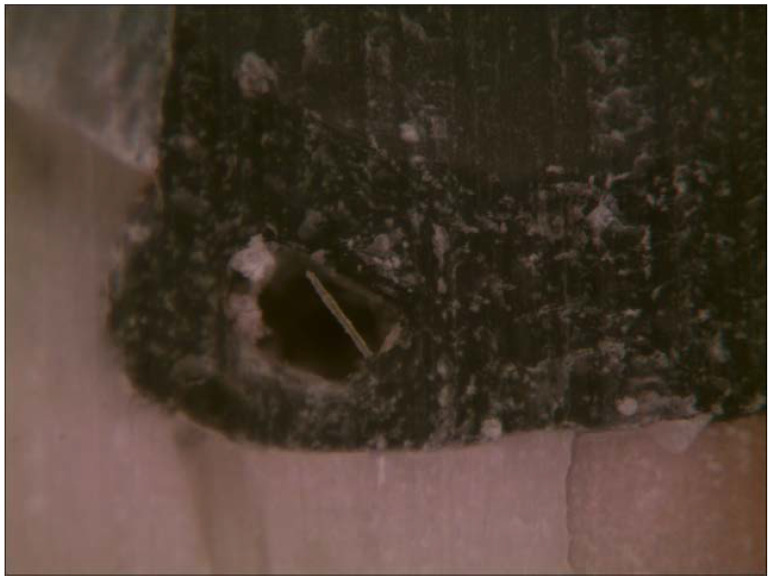
Internal air-bubble formation within the cured SFRC material.

Therefore, during polymerization, the material cannot shrink along the length of the fibres and retains its original dimensions horizontally, while the polymer matrix between the fibres can shrink [35]. In many specimens, internal bubble formation within the material could be seen when SFRC was used (Figure 5).

The same was observed by clinicians when using packable SFRC in a bulk manner. As suggested by Fráter and co-workers, these bubbles could be partly a sign of internal stress relief: if shrinkage does not occur at the interface, it might cause internal voids inside the bulk material [67].

The continuity of the developed gaps was measured with dye penetration in this study. Teeth restored with SDR showed shallower dye penetration along the interphase and also showed the highest proportion of perfect margins. According to Peutzfeldt et al., whether marginal gaps are formed depends on an interplay between multiple factors, of which some are related to the resin composite, while others are related to the specific cavity and restorative procedure [4]. In our study, we sought to keep the factors related to the cavity and the restorative procedure constant. Standardized cavities were prepared to ensure that all cavities had a high C-factor. Our finding regarding the superiority of SDR in terms of marginal integrity is in line with the findings of de Assis et al. [50] and Gerula-Szymańska et al. [68], but they contradict the findings of de Dietschi et al. [69]. When looking at the number of continuous gaps in Group 1 (layered composite filling) and Group 3 (SFRC), both groups produced deeper penetration, even many times reaching the floor of the cavity (Figure 5, Table 5).

One of the limitations of our study is that static load-to-fracture testing was used without fatigue testing. While static load-to-fracture tests model a sudden, greater force (such as when trauma occurs), dynamic loading is more appropriate to study the mechanical consequences of the forces that act during ordinary chewing. An accelerated dynamic loading test represents a realistic compromise between the two extremes. In this test, cyclic loading is applied, but the magnitude of the force is not constant for the entire duration of the test: it increases stepwise, always after a given number of cycles have been completed [70]. Thus, fatigue testing should be carried out in the future in the same groups. The other limitation of this study is that the specimens were not thermocycled before the marginal integrity analyses. As shown by Dietschi et al. [69], adaptation can greatly differ before and after thermocycling. Our results should be interpreted with this limitation in mind. Future studies should incorporate thermocycling, fatigue testing and also other bulk-fill materials to better understand the above proposed research topic.

## 5. Conclusions

Bulk-fill resin composites (SDR and SFRC) have demonstrated encouraging results in terms of fracture resistance when compared to conventional packable composite. Furthermore, these bulk-fill materials showed improved internal adaptation to the cavity walls compared to the gap formation present in case of utilising conventional composite material in a high C-factor cavity. Moreover, SFRC showed more restorable fracture behaviour compared to the other tested materials. Thus, bulk-fill materials provide a simple and effective solution for restoring and reinforcing high C-factor occlusal cavities.

## Figures and Tables

**Table 1:** Restorative materials used during the study.

Category	LOT Number	Material	Manufacturer	Composition
Conditioner	BFDJ8	Ultra-Etch	Ultradent	35% phosphoric acid, water, cobalt aluminate blue, spinel, glycol, siloxan
Adhesive	1703031	G-Premio Bond	GC	10-MDP (5–10%), 4-MET, dimethacrylate (10–20%), dimethacrylate component (1–5%), photo-initiator (1–5%), butylated hydroxytoluene (<1%), acetone (25–50%), water (24%)
Composite	N841976	Filtek Ultimate Composite Resin	3M	Bis-GMA, UDMA, TEGDMA, Bis-EMA, 20 nm silica and 4–11 nm zirconia filler, camphorquinone, accelerators, pigments and others.
SFR composite	1212261	EverX Posterior	GC	Bis-GMA, PMMA, TEGDMA, 74.2 wt%, 53.6 vol% Short E-glass fibre filler, barium glass
Bulk-fill composite	1202174	Surefil SDR	Dentsply	TEGDMA, EBADMA, 68 wt%, 44 vol%, Barium borosilicate glass

SFR: Short-fibre reinforced; Bis-GMA: 2,2-bis[p-(2-hydroxy-3-methacryloxy propoxy)phenyl]propane; MDP:10-methacryloyloxydecyl dihydrogen phosphate; 4-MET: 4-methacryloyloxyethyl trimellitate; MEPS: methacryloyloxyalkyl thiophosphate methylmethacrylate; TEGDMA: triethylene glycol dimethacrylate; UDMA: urethane dimethacrylate; PMMA: polymethyl methacrylate, PEGDMA: poly (ethylene glycol) dimethacrylate; EBPADMA: Ethoxylated bisphenol A dimethacrylate.

**Table 2:** Physical properties of the restorative materials, according to the manufacturers.

Material	Volumetric Shrinkage (%)	Fracture Toughness (MPa m^1/2^)	Flexural Strength (MPa)
Filtek Ultimate Composite Resin	2.0	1.22	160 ± 20
EverX Posterior	2.9	2.61	153 ± 9
Surefil SDR	3.5	1.25	120 ± 13

**Table 3:** Four grade scale showing degree of microleakage.

Score	Content
0	No Microleakage
1	Dye penetration within the occlusal half of the axial cavity wall
2	Dye penetration extending into the lower half of the axial cavity wall
3	Dye penetration spreading along cavity floor

**Table 4:** The distribution of fracture patterns among the tested groups (*n* = 12).

Study Group	Restorable Fracture	Non-Restorable Fracture
Group 1 (Composite)	7	5
Group 2 (SDR)	2	10
Group 3 (SFRC)	12	0

**Table 5:** The microleakage score among the tested groups (*n* = 12).

Dye Penetration Score	Group 1 (Composite)	Group 2 (SDR)	Group 3 (SFRC)
0	1	4	2
1	4	7	3
2	6	1	5
3	1	0	2

## Data Availability

Data is contained within the article.
